# Contribution of the Bony Bankart in Calculating Glenoid Bone Loss

**DOI:** 10.1177/23259671231168879

**Published:** 2023-05-18

**Authors:** Isabella Bozzo, Paul Kooner, Ralph Nelson, Yousef Marwan, Samir Paruthikunnan, Carl Laverdière, Mathieu Boily, Paul A. Martineau

**Affiliations:** † Faculty of Medicine, McGill University, Montréal, Québec, Canada; ‡ Division of Orthopaedic Surgery, McGill University Health Centre, Montréal, Québec, Canada; § Department of Diagnostic Radiology, McGill University Health Centre, Montréal, Québec, Canada; || Department of Surgery, Faculty of Medicine, Health Sciences Center, Kuwait University, Kuwait City, Kuwait; ¶ York and Scarborough Teaching Hospitals NHS Foundation Trust, York, UK

**Keywords:** shoulder, instability, imaging computed tomography, general sports trauma, Bankart fracture

## Abstract

**Background:**

Determining the magnitude of glenoid bone loss in patients with anterior shoulder instability is an important step in guiding management. Most calculations to estimate the bone loss do not include the bony Bankart fragment. However, if it can be reduced and adequately fixed, the estimation of bone loss may be decreased.

**Purpose:**

To derive a simple equation to calculate the surface area of the bony fragment in Bankart fractures.

**Study Design:**

Case series; Level of evidence, 4.

**Methods:**

A total of 26 patients suspected of having clinically significant bone loss underwent computed tomography imaging preoperatively, and the percentage of glenoid bone loss (%BL) was approximated with imaging software using a freehand region of interest area measurement with and without the inclusion of the bony Bankart fragment. By assuming this bony fragment as a hemi-ellipse with height, H, and thickness, d, we represented the surface are of the bony piece ( $A_{\mathrm{bone}\;\mathrm{fragment}}=\frac{\pi \mathrm{Hd}}{4}$ ), and subtracted it from the overall %BL. They compared this value with the one found using imaging software.

**Results:**

Without the inclusion of the bony Bankart, the overall %BL by the standard true-fit circle measured using imaging software was 23.8% ± 9.7%. When including the bony Bankart, the glenoid %BL measured using imaging software was found to be 12.1% ± 8.5%. The %BL calculated by our equation with the bony Bankart included was 10% ± 11.1%. There was no statistically significant difference between the %BL values measured using the equation and the imaging software (*P* = .46).

**Conclusion:**

Using a simple equation that approximates the bony Bankart fragment as a hemiellipse allowed for estimation of the glenoid bone loss, assuming that the fragment can be reduced and adequately fixed. This method may serve as a helpful tool in preoperative planning when there are considerations for incorporating the bony fragment in the repair.

Determining the magnitude of glenoid bone loss in patients with anterior shoulder instability is an important step in guiding management for shoulder surgeons. A large deficit of the glenoid width has been associated with poor outcomes after arthroscopic repair and is often an indication for a more extensive bony reconstruction.^3^ Therefore, a simple and accurate calculation of the degree of glenoid bone loss is helpful for determining the most successful treatment option. Many studies have developed methods to quantify bone loss using preoperative imaging or intraoperative measurements; however, none have considered the contribution of including the osseous bony Bankart lesion to restoring glenoid bone loss.

A majority of large glenoid defects after shoulder instability have a bony fragment of variable size near the anteroinferior portion of the glenoid neck.^15^ Sugaya et al^15^ found that in a series of 42 patients with recurrent shoulder instability, all patients had a fragment near the anteroinferior glenoid. These fragments are often connected firmly to adjacent capsule or labrum and have been reported even in cases with bone loss. While no true gold standard exists in calculating glenoid bone loss, we propose a simple calculation with the inclusion and contribution of the bony fragment to the glenoid surface area.

Currently, there are many described methods to calculate glenoid bone loss after shoulder instability.^1,4,7^ The most popular technique is the “true-fit circle” method, in which a computed tomography (CT) en face view of the glenoid is used to determine the area of the true-fit circle (*A*) and the area of presumed bone loss (*B*), with the ratio (*B*/*A*) × 100 giving the percentage of glenoid bone loss (%BL).^2,15^ Multiple variations of this technique are reported in the literature using several imaging techniques including CT, 3-dimensional reconstructed CT, or magnetic resonance imaging scans on both affected and unaffected sides to determine true glenoid deficiency.^1,4,7^

While no universal consensus exists on measuring techniques, no technique considers the osseous bony fragment in the calculation of the defect. With the advancement of arthroscopic surgical techniques and the increased recognition of the bony contribution to stability, specific techniques, such as the use of a suture bridge fixation, may be used to reduce this piece to the glenoid. We propose a simple and clinically applicable calculation of %BL with the inclusion of the bony fragment in the equation. By incorporating this piece, we may decrease our overall estimation of bone loss.

The purpose of this study was to calculate glenoid bone loss using a clinically applicable equation incorporating the bony Bankart fragment to the overall calculated glenoid surface area. We compared this value with the standard calculation of bone loss without the fragment and the calculation with more involved radiological area measurement methods.

## Methods

Research ethics board approval for this study was obtained. We retrospectively reviewed the records of patients who underwent surgery for shoulder instability from June 1, 2008, to December 31, 2019. The study inclusion criteria were patients aged >18 years who underwent surgery for shoulder instability and who had preoperative CT scans. Preoperative CT scans were performed in patients who were suspected of having clinically significant bone loss from physical examination and preoperative radiographs to better characterize the bone loss and aid in surgical planning. Exclusion criteria included patients with concomitant bony pathologies, such as clavicular, coracoid, or proximal humeral fractures in the acute setting; a history of previous fractures in the assessed shoulder; or metabolic/genetic bone diseases.

### Derivation of Geometric Glenoid Bone Loss Equation

**Figure 1.**
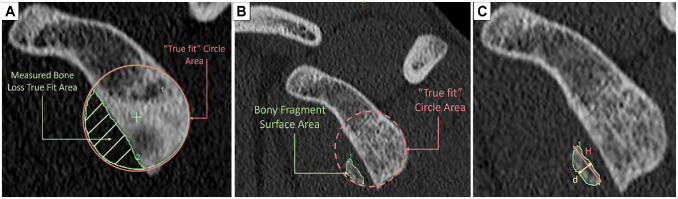
Computed tomography images with schemas of the relevant anatomy and surface areas of the en face view of the glenoid. (A) Glenoid bone head with the measured true-fit circle area and the area of bone loss approximated using imaging software. (B) En face view of the bony Bankart fragment with surface area approximated using imaging software. (C) En face view of the bony Bankart fragment with surface area approximated using imaging software and with labeled dimensions for Equation 1, where *H* is the length of the flat edge of the bony fragment and *d* is the thickness of the bony fragment along the traverse plane.

The primary outcome of this study was to apply a new equation to calculate the %BL based on a geometric model of the glenoid surface area including the osseous bone fragment. The dimensions were obtained for preoperative CT scans with an en face view of the glenoid surface. The first mathematical assumption was that the surface area of the glenoid cavity may be approximated by a true-fit circle, as shown in Figure 1A. The second was that the bony Bankart fragment could be approximated by a hemiellipse, as shown in Figure 1B.

With these assumptions, we calculated the following equations:

Equation 1. Area (*A*) of the bony fragment (approximated as a hemiellipse, *H* = height, *d* = width) (Figure 1C): $A_{\mathrm{bone}\;\mathrm{fragment}}=\frac{\pi \mathrm{Hd}}{4}$ .

Equation 2. %BL with true-fit circle, no bone fragment: $\frac{A_{\mathrm{measured}\;\mathrm{bone}\;\mathrm{loss}}}{A_{\mathrm{true}-\mathrm{fit}\;\mathrm{circle}}}\times 100\%$ .

Equation 3. %BL with true-fit circle, with fragment: $\frac{A_{\mathrm{measured}\;\mathrm{bone}\;\mathrm{loss}}\;-\;A_{\mathrm{bone}\;\mathrm{fragment}}}{A_{\mathrm{true}-\mathrm{fit}\;\mathrm{circle}}}\times 100\%$ .

### Measurement Protocol

Two independent raters, a musculoskeletal fellowship trained radiologist (M.B.) and a musculoskeletal radiology fellow (S.P.), reviewed all CT scans that had an en face view of the glenoid. The %BL found by our equation was compared with the values found using imaging software InteleViewer 4.14.1 (Intelerad Medical Systems Inc) to directly calculate the surface area of a selected geometry using freehand region of interest area measurement. The calculated glenoid bone loss with and without the fragment was compared with the surface area as measured by the imaging software. The radiologists were instructed to measure the dimensions of the (1) glenoid head and (2) bony fragment from 2 separate en face CT slices that would independently maximize the area of each component, since the fragment may be displaced medially. Postoperative outcomes were not assessed in this study, which focused only on the procedures patients should be indicated for preoperatively based on the amount of glenoid bone loss.

Statistical analysis was performed using SPSS (IBM Corp) with 2-tailed *t* tests and calculations of means with standard deviations to compare these values.

## Results

There were 50 patients who underwent surgery for shoulder instability who were suspected of having significant bone loss and underwent preoperative CT imaging at a university health care center over 12 years. Of the 50 patients, 26 patients fully met the inclusion criteria, including the presence of bony Bankart fragments on preoperative CT imaging. The mean patient age was 35.3 ± 14.7 years (range, 18-75 years), and the male/female ratio was 25:1.

### Calculation of Glenoid Bone Loss With Incorporation of the Bony Bankart

**Figure 2.**
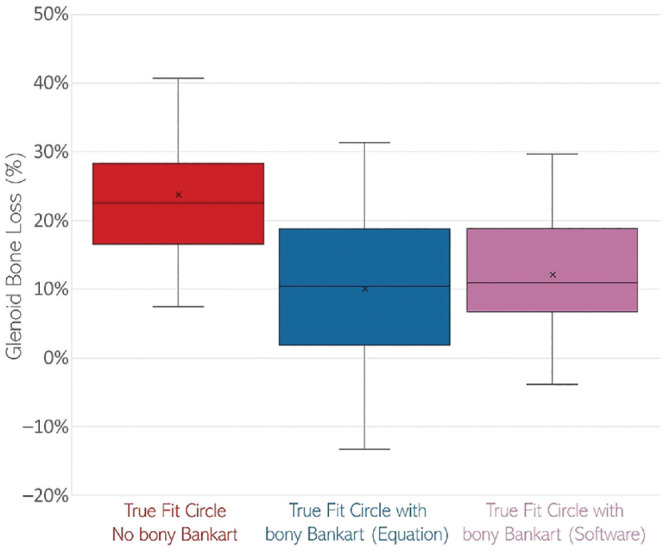
Glenoid bone loss calculated with the true-fit circle without the inclusion of the bone fragment surface area (red), with inclusion of the bone fragment surface area (calculated with equation; blue), and with inclusion of the bone fragment surface area (measured using imaging software; purple). The top and bottom bars represent the total range of the data, i.e., the largest and smallest values in the dataset respectively. The middle line represents the median value. The X represents the mean value. The box represents the range for 2 SD (standard deviations) of the 2-tailed Student’s t-test.

A summary of the percentage bone loss calculations with and without the bone fragment for each patient is shown in Table 1. Without the inclusion of the bony Bankart, the overall mean %BL by the standard true-fit circle measured using the imaging software was 23.8% ± 9.7%. When including the bony Bankart, the mean %BL calculated using imaging software was found to be 12.1% ± 8.5%. The %BL calculated by our equation with the bony Bankart included (Equation 3) was 10% ± 11.1%. There was no statistically significant difference between the %BL values with our equation and the imaging software, as shown in Figure 2 (*P* = .464).

**Table 1:** Glenoid Bone Loss Per Patient, Measured With the True-Fit Circle Area Method With and Without the Bony Fragments Included in the Reconstruction Surface Area

	Bone Loss, %			Reduction in Bone Loss, %	
Patient No.	Without Bone Fragment	With Bone Fragment Measured Using Imaging Software	With Bone Fragment Calculated With Equation	Measured Using Imaging Software	Calculated With Equation
1	23.67	18.03	19.28	5.64	4.39
2	20.88	19.10	19.29	1.78	1.59
3	38.56	14.89	17.32	23.67	21.24
4	21.48	1.10	−4.39	20.37	25.86
5	25.09	−3.86	−13.31	28.95	38.39
6	27.05	6.18	3.24	20.87	23.81
7	32.23	11.14	−4.54	21.09	36.77
8	27.27	0.95	−6.50	26.32	33.78
9	49.62	26.14	31.32	23.48	18.30
10	28.67	10.83	1.38	17.83	27.29
11	30.13	24.34	21.38	5.79	8.75
12	20.62	8.47	8.93	12.15	11.69
13	14.93	11.01	11.17	3.91	3.76
14	14.40	10.88	10.7	3.52	3.70
15	33.00	23.57	24.02	9.43	8.98
16	27.10	21.37	21.56	5.73	5.54
17	16.41	9.85	10.44	6.56	5.97
18	40.73	29.70	28.87	11.02	11.86
19	17.07	11.10	10.13	5.97	6.94
20	18.52	0.15	−2.88	18.37	21.40
21	13.50	10.24	10.36	3.25	3.14
22	24.73	19.27	16.95	5.46	7.78
23	15.81	8.32	5.71	7.49	10.10
24	21.10	14.81	15.33	6.28	5.77
25	7.48	5.43	4.71	2.05	2.77
26	8.10	2.12	0.87	5.98	7.23
Mean	23.77	12.12	10.05	11.65	13.72

The mean percentage reduction in bone loss using our equation and including the bony fragment was 13.7%. This was comparable with the true percentage reduction measured using the imaging software (*P* = .458). A post hoc power analysis (2-tailed Student *t* test with matched pairs) demonstrated sufficient power, and the error between the equation and the more involved area method was not statistically significant (mean, 2.97 ± 3.7; effect size, 0.8; alpha, .05; and power, 0.8).

## Discussion

In this study, we present a simple and clinically relevant equation to characterize the degree of bone loss with the inclusion of the bony fragment of the glenoid, which is often found displaced near the fossa. If it is assumed that the fragment can be reduced and fixed, then the resulting estimated overall glenoid bone loss is reduced.

The true-fit circle method is a common method for estimating the degree of glenoid bone loss. Further evaluation of this method shows that it can overestimate this value, leading to compromised clinical judgment. Bhatia et al^2^ compared this diameter-based calculation with a true value found with imaging software and concluded it can overestimate bone loss as much as 20% from the actual value. This is consistent with other findings in the literature, as Piasecki et al^12^ found an average overestimation of 5.8%. While our equation only modifies this method, we found that by adding the bony fragment and assuming it to be a hemiellipse in our calculation ( $A_{\mathrm{bone}\;\mathrm{fragment}}=\frac{\pi \mathrm{Hd}}{4}$ ), we can consistently quantify the degree of bone loss with this piece when compared with the measurements with imaging software.

There is a paucity of literature examining the influence of incorporation of the bony Bankart fragment on glenoid defect size after shoulder instability surgery. Recent literature has recognized the integrity of the bony fragment and its contribution to decreasing glenoid defect size before definitive surgical procedures. Sugaya et al^14^ found that arthroscopic repair of bony Bankart fragments using suture anchors was successful because most of the bone fragments were found to be preserved, even in chronic glenoid defects. Furthermore, Nakagawa et al^10,11^ found that after bony Bankart repair, glenoid defect size decreased significantly compared with preoperative defect size. Further studies have shown that glenoid rim morphology has even enlarged after bony union of this fragment because of remodeling.^8,14^ Accordingly, our simple calculation will allow surgeons to accurately estimate bone loss with the incorporation of this fragment preoperatively, if reduction of the bony fragment is being considered, before determining the final glenoid defect size.

Controversy exists over the critical threshold value of glenoid bone loss, which may lead to indicating one treatment option versus another. Lo et al^9^ argued for bony reconstruction when there was >25% glenoid deficiency, showing an unacceptably high failure rate after soft tissue procedures. Recent literature has suggested that this threshold should be lower, with studies recommending 13.5% bone loss as an indication for bony reconstruction.^5,13^ While such debate exists over deciding on the most optimal treatment, we feel this gives even more reason to consider including the osseous bone fragment if present. To assess the potential clinical relevance, the amount of bone loss was compared with critical bone loss cutoff values, either 13.5% or 25%, as these are the extremes cited in the literature and used in clinical practice. With a critical threshold value of 13.5%, if the bone fragment was reduced arthroscopically in the repair, this would have changed the indication of surgical treatment of 50% of our cohort (13 of the 26 patients) in favor of soft tissue repair. While at a cutoff value of 25%, 9 of the 26 patients would have been indicated for a soft tissue repair.

Soft tissue stabilization procedures were not evaluated in this study, and this is an important limitation of bony arthroscopic repairs, as they do not account for glenohumeral ligament elongation. Arthroscopic and open rotator interval plications have been proposed as an adjunct to bony repairs to reduce recurrence rates; however, there are concerns for loss of external rotation postoperatively that need to be further studied.^6^ These outcomes are quite significant, as it is important to consider the additional risks of more complex bone augmentation procedures versus soft tissue repair.

### Limitations

This study has several limitations. First, our equation assumes that there is an osseous bone fragment present on imaging and that it is represented by a hemiellipse. This may not always be the case, as it sometimes varies in size with bone erosion over time. Second, several parameters should be considered for the surgical decision-making process other than bone loss, including age, activity level, and humeral-sided bone loss. While critical threshold values are important, other patient factors must be considered while making clinical judgment. Third, with a relatively small sample size, only patients suspected of having clinically significant bone loss from clinical examination and preoperative radiographs, who then underwent preoperative CT imaging to characterize the bone defect, were selected in this study and thus represent only severe cases of bone loss. Last, with no clinical outcomes to follow, future indications include patient outcomes to correlate with this analysis. Moving forward, future work will present the postoperative outcomes of patients who undergo repair with arthroscopically reduced bony fragments compared with isolated arthroscopic soft tissue repairs and open bony procedures.

## Conclusion

In this study, a simple equation that approximates the area of the bony Bankart fragment as a hemiellipse allowed for estimation of the %BL by assuming that the fragment can be reduced surgically and adequately fixed. This method may serve as a helpful tool in preoperative planning when there are considerations for incorporating the bony fragment in the repair.
